# MicroRNA-146a Contributes to SCI Recovery via Regulating* TRAF6* and* IRAK1* Expression

**DOI:** 10.1155/2016/4013487

**Published:** 2016-10-17

**Authors:** Jinsong Wei, Jiafeng Wang, Yulan Zhou, Shouquan Yan, Keshen Li, Hongsheng Lin

**Affiliations:** ^1^Department of Orthopedics, The First Affiliated Hospital of Jinan University, Guangzhou, China; ^2^Stem Cell Research and Cellular Therapy Center, Affiliated Hospital of Guangdong Medical University, Zhanjiang, China; ^3^Institute of Neurology, Affiliated Hospital of Guangdong Medical University, Zhanjiang, China; ^4^Stroke Center, Neurology & Neurosurgery Division, The Clinical Medicine Research Institute and The First Affiliated Hospital of Jinan University, Guangzhou, China

## Abstract

MicroRNA-146a participates in spinal cord injury (SCI) recovery. Until recently, how miRNA-146a participates in SCI remained unclear. In this study, we tried to explore the roles of miRNA-146a in the recovery of SCI using a rat model. The expression of the probable target genes of miRNA-146a (including IRAK1 and TARF6) as well as proinflammation cytokines were measured until 7 days after surgery in the three groups (sham group, SCI group, and miRNA-146a antagomir injection group). Also, the animals' motivations were estimated using Basso Beattie Bresnahan (BBB) during the whole experiment. A luciferase assay was performed to demonstrate that miRNA-146a could directly target the mRNAs of* IRAK1* and* TRAF6*. Our experiments indicate that miRNA-146a inhibits proinflammatory cytokine secretion by suppressing* IRAK1 *and* TRAF6 *expression in the SCI model. In contrast, miRNA-146a may be upregulated by inflammatory mediators via the* IRAK1*/*TRAF6* pathway in the spinal cord. As a negative feedback element, miRNA-146a could make sure that the expression of* IRAK1*- and* TRAF6*-mediated genes was under tight control. Thus, miRNA-146a may serve as a novel therapeutic target for SCI interventions.

## 1. Introduction

Spinal cord injury (SCI) is one of the most common injuries that is observed in spine and neurosurgery departments. The spinal cord is very susceptible to injuries and its self-repair capacity is limited. SCI is usually caused by sports injuries, building accidents, motor vehicle accidents, and violence. SCI can cause paralysis or loss of movement or sensation. Currently, the treatment of SCI remains one of the greatest challenges for clinical investigators, and the understanding of mechanisms that can be utilized by SCI therapeutics is still limited.

Biochemical disturbances accompany vascular alterations and cellular responses that are caused by SCI which will activate the inflammatory response. After that, cell apoptosis, Wallerian degeneration, and scarring will occur from days to years after the injury [1, 2].

Other investigators have previously reported that different gene expression along with posttranscriptional regulation significantly contributes to SCI pathogenesis [1, 3, 4]. Meanwhile, miRNAs have recently become the most important posttranscriptional regulators due to their ability to inhibit mRNA translation [5]. miRNA is a kind of noncoding small RNA, which is usually no longer than 22 nucleotides. The miRNAs are able to regulate gene expression by destabilizing mRNA translation [6, 7]. Individual miRNAs may affect a biological process by coordinating the translation of several even dozens of coding genes [8].

More and more evidence that miRNAs are involved in immune response have been found in recent studies [9, 10]. Furthermore, there is now accumulating evidence suggesting that miRNA-146a participates in the regulation of the innate immune response, which is well known for its regulation of the TLR signaling pathway and proinflammatory cytokines [11–13]. miRNA-146a has been certified to downregulate its target genes TNF receptor-associated factor 6 (*TRAF6*) and IL-1 receptor-associated kinase 1 (*IRAK1*), which leads to the inhibition of the inflammatory reaction in macrophages, monocytes, astrocytes, and neurons [14–17]. Also, Lu et al. [18] demonstrated that miRNA-146a could attenuate neuropathic pain by suppressing* TRAF6* signaling in the spinal cord.

Preliminary studies using next generation sequencing (NGS) examined the miRNA expression profiles post-SCI in animal models. Increasing expression of miRNA-146a was found in animal SCI models [5, 8]. Another study examined miRNA expression profiles post-SCI in rats using microarrays [19], which confirmed that there were significant and common changes in miRNA-146a expression. However, there is currently limited information regarding the role of miRNA-146a and its mechanism of action. The putative role of miRNA-146a in SCI is still based on in silico predictions, and no direct evidence is reported. How miRNA-146a modulates the downstream target genes in SCI is still unknown, given that* TRAF6 *and* IRAK1 *play an important role in neuroinflammation [20, 21], and miRNA-146a acts as a key regulator of the inflammatory reaction [15, 16]. In this study, we determined the role of miRNA-146a in SCI and tried to find out how it participated in SCI recovery by regulating its target genes (*TRAF6* and* IRAK1*) using a rat SCI model.

## 2. Material and Methods

### 2.1. Animals Used

Adult, female Wistar rats weighing approximately 200 g obtained from the Laboratory Animal Center of Guangdong Medical University (Guangdong, China) were used for all experimental procedures. The rats were housed in wire cages (50 × 30 × 20 cm) with food and water continuously available.

### 2.2. SCI Model and Treatment

A rat SCI model was established by compressing the spinal cord using a forceps with a preestablished separation of the blades for 10 sec (approximately 200 kdyne, which was determined by preliminary experiments) at the 8~10th thoracic vertebra to induce a spinal cord injury. Forceps blade separation was confirmed before each surgery with microcalipers making sure that the dura remained intact during injury introduction. After compressing, the injury site was washed with room temperature saline. Most SCI rats exhibited flaccid paralysis or spasm of the lower extremities after the SCI surgery. In our study, the rats that exhibited flaccid paralysis of the lower extremities were chosen for the next study. The rats' bladders were manually expressed to assist urination twice a day until the animals regained bladder control. The injured animals were evaluated for lower extremities locomotion using BBB scale.

After SCI surgery, the rats were divided into 3 distinct groups: one group was injected with miRNA-146a antagomir negative control (SCI + NC), one group was injected with miRNA-146a antagomir (SCI + antagomir), and the last group was given laminectomies without the injection (sham). The tail intravenous injection was used for all the animal injections. After surgery, the rats were housed individually, and long sipper tubes were used to make sure that the rats could reach the water freely. The animals were anesthetized with an intraperitoneal injection of 7% chloral hydrate at 75 mg/kg before the animals were sacrificed.

### 2.3. RNA and Protein Extraction

Spinal cord fragments around the injury were cut out from the vertebra after the rats were sacrificed. Then, the fragments were maintained in RNAlater buffer (Qiagen) until total RNA extraction. Another group of animals treated in parallel was used for histopathology.

Total RNA was extracted by SV Total RNA Isolation (Promega USA), and the concentration and purity were determined using a Nanodrop2000 spectrophotometer. RNA integrity was determined using agarose gel electrophoresis.

### 2.4. miRNA-146a Inhibition

**Figure 1:**
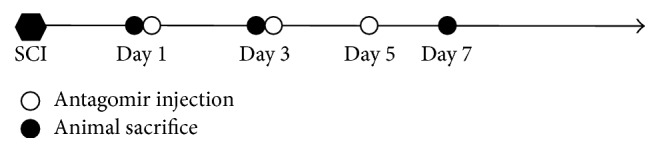
Schematic flowchart of the experimental procedure for SCI treatment and evaluation. 10 animals were involved in each treatment (three animals each time were sacrificed at day 1, day 3, and day 7 after SCI, one more extra animal prepared for in case of animal accidental death).

MicrOFFTM mmu-miRNA-146a antagomir (RIBOBIO, Guangzhou, China) acted as a high-efficiency antagonist for miRNA-146a and was used for inhibiting the expression of miRNA-146a. Because the efficacy of the antagomir produced by RIBOBIO can last for two weeks in animal bodies, antagomir (5 nmol) was injected into animal bodies through the tail vein to avoid the extra injury from intrathecal injection on day 1, day 3, and day 5 after surgery. The time axis of injection and animal sacrifice are illustrated in Figure 1.

### 2.5. Real-Time Quantitative PCR (qPCR) and WB (Target Genes)

MRNA reverse transcription was performed using an oligo dT primer and MMLV enzyme according to the manufacturer's protocol. A small RNA extraction kit (Takara) was used for miRNA detection, and the small RNA reverse transcription was performed using PrimeScript miRNA cDNA Synthesis Kit (Takara). Real-time quantitative PCR analysis was performed using Light Cycler® 480(Roche) with SYBR green dye detection (Takara). The primers used in our study were summarized as shown in Table 1.

Thermal cycle of qPCR amplifications was performed at 95°C for 1 min, followed by 40 cycles at 95°C for 5 s and 60°C for 45 s, and a 10 min incubation at 72°C was carried out at the end of the cycles. GAPDH and U6 were adopted as endogenous controls to normalize differences for mRNA and miRNA detection, respectively. Quantification analysis using normalizing cycle threshold values (Ct) with GAPDH Ct or U6 Ct and the data was analyzed using the 2^−ΔΔCT^ method. Melt curves were also performed after the amplification to avoid nonspecific products.

The protein concentrations were determined using a BCA protein assay kit (Shanghai, China) after the spinal cord was harvested. Western blotting analyses were performed using SDS-PAGE. After PAGE, the proteins were transferred to PVDF membranes. Then, the membranes were blocked in 5% nonfat milk for 1.2 h at room temperature and then incubated with antibodies overnight at 4°C. Antibodies against* IRAK1* and* TRAF6* were obtained from PL Laboratories (Canada), and the antibody against *β*-actin was purchased from Sigma. Proteins were visualized with enhanced chemiluminescence. Relative intensities were determined using Quantity One 4.6.2 software (Bio-Rad, USA), and GAPDH was used as the internal control. Data were given as the mean ± SD of the percentage ratio of the control.

### 2.6. Cytokines Detection

The inflammatory cytokines (including TNF-*α*, IL-1*β*, IL-6, IL-12, and RANTES) levels were determined to examine the effects of miRNA-146a on the levels of cytokines at the injured region. Spinal cord sample around the injury site was dissected and incubated in cell medium DMEM (Sigma) supplemented with 10% FBS and 0.2% paramucin. Inflammatory cytokines were detected after a 24 h incubation using a milliplex inflammatory cytokine kit (Millipore, USA). The cytokines were incubated with the antibodies in Elisa plates, after washing the extra antibodies, the absorbance was measured using a multiscan spectrum.

### 2.7. Luciferase Assay

In order to confirm whether miRNA-146a could directly target the mRNAs of* IRAK1* and* TRAF6*, the 3′-UTR sequence of* IRAK1* and TRAK6 was cloned into the 3′ site of the luc2 reporter gene on a plasmid (Promega, USA) to construct a report plasmid. Then, 300 ng of the plasmid, 30 ng of pRL-TK-Renilla luciferase, and the miRNA mimic (final concentration 40 nM) were cotransfected in SH-SY5Y using Lipofectamine 2000. Luciferase activity was measured by the Dual-Luciferase Reporter Assay (Promega) according to the manufacturer's protocol 24 hours after transfection.

### 2.8. Recovery of SCI and BBB

The animals were acclimated to the outside fields for 3 days befor surgery to avoid subjects holding still (i.e., freezing) when they are introduced to a new apparatus. The BBB scale [22] was used for assessing locomotor behavior for 7 days after surgery in an open enclosure. Each animal was placed alone in the open field for 5 min, and locomotor behavior scores were derived according to the procedure developed by Basso et al. [22], making sure that the investigators' scoring behavior had high intra- and interobserver reliability, and the person that provided the score was blind to the subject's experimental treatment. All the scores were acquired three times to make sure the data were accurate.

### 2.9. Statistical Analysis

The results represent the mean ± standard deviation (SD). Differences in the data were tested for statistical significance using two-way ANOVA or one-way ANOVA. For all tests, *P* < 0.05 was considered to be statistically significant.

## 3. Results

### 3.1. Overexpression of miRNA-146a in the Animal Model of SCI

**Figure 2:**
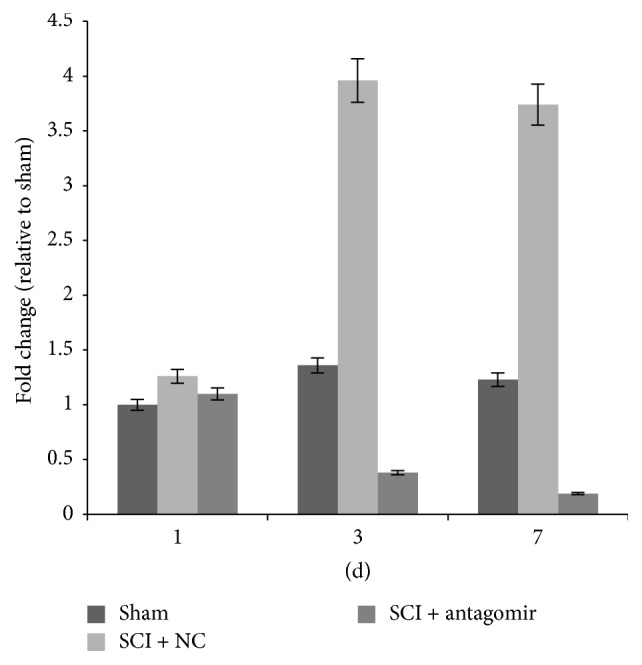
Expression of miRNA-146a in different model groups. Three replications in each treatment of each time point; the average expression level was adopted.

In the negative control (SCI + NC) group, miRNA-146a was increased (*P* < 0.05), whereas it was obviously downregulated after the miRNA-146a antagomir injection on the third and seventh day after surgery (Figure 2). Remarkable upregulation of miRNA-146a was not detected on the first day after surgery in the SCI + NC group, and also there was no difference in the expression of miRNA-146a during the experiment in the sham group (Figure 2).

### 3.2. *IRAK1* and* TRAF6* Were Regulated by miRNA-146a

**Figure 3:**
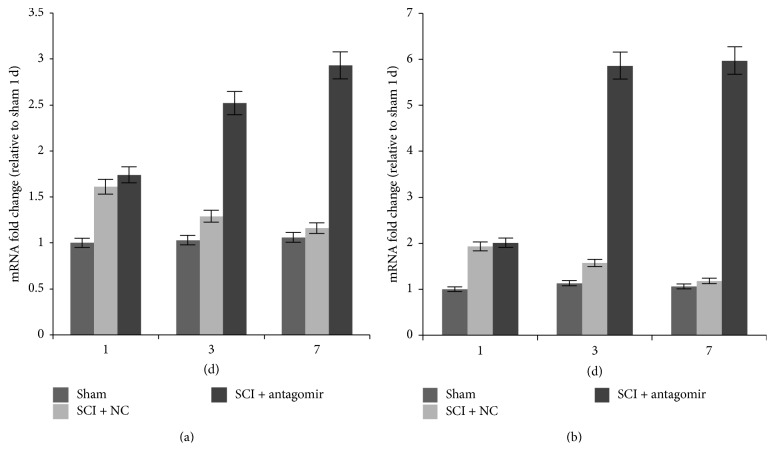
mRNA levels of the target genes in different model groups. (a) TRAF6 mRNA levels and (b) IRAK1 mRNA levels. Three replications in each treatment of each time point; the average expression level was adopted.

**Figure 4:**
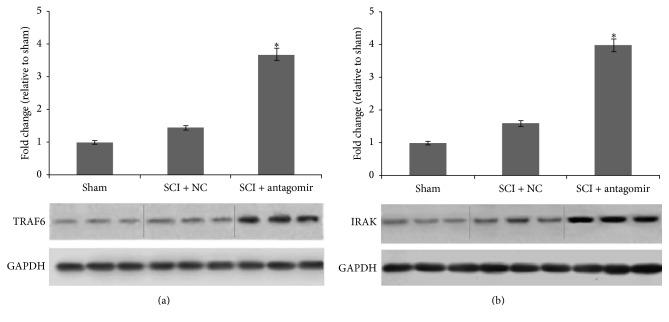
Protein levels of the target genes in different model groups 7 days after SCI. (a) TRAF6 protein levels and (b) IRAK1 protein levels. Three replications in each treatment of each time point; the average expression level was adopted; *∗* denotes the significant difference, *P* < 0.05.

Compared to miRNA-146a, the expression of genes* IRAK1* and* TRAF6* presented the opposite regulation model (Figure 3). The transcription of the two genes was increased on the first day after surgery and then decreased significantly over the next couple of days in the SCI + NC group. However, in the SCI + antagomir injection group, there was a dramatic upregulation of* IRAK1* and* TRAF6* in mRNA transcription on days 3 and 7 after SCI. Similarly, significant protein levels of the two genes were also detected and rose significantly in the antagomir injection group compared to the sham or SCI + NC group 7 days after the operation (Figure 4).

**Figure 5:**
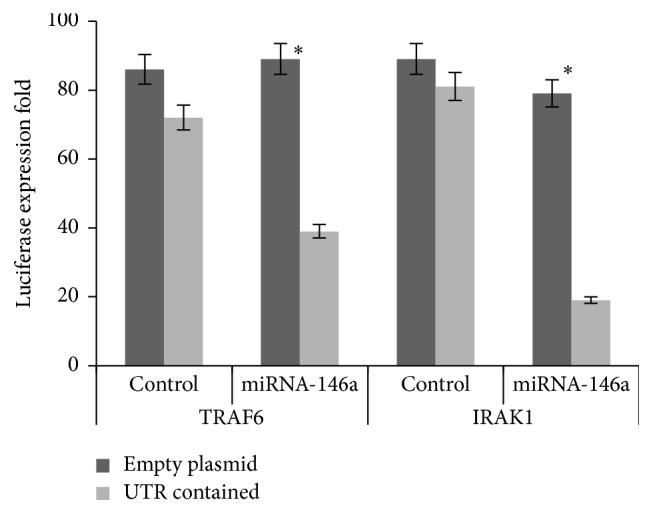
Luciferase levels comparison of plasmids contained the (IRAK1 and TRAF6) UTR and the control group. The expression levels are shown as the mean ± SD; *∗* denotes the significant difference, *P* < 0.05.

In order to detect a direct interaction between miRNA-146a and the* IRAK1*/*TRAF6* 3′-UTR, we, respectively, cloned the* IRAK1*/*TRAF6* 3′-UTR sequence downstream of the luciferase reporter gene. In the luciferase assay, the miRNA-146a mimics reduced the luciferase levels in the UTR-containing plasmids compared to the control group (*P* < 0.05; Figure 5). These results suggested that miRNA-146a may modulate* IRAK1* and* TRAF6 *expression by directly targeting the 3′-UTR of their mRNA.

### 3.3. Expression of Proinflammatory Cytokines

**Figure 6:**
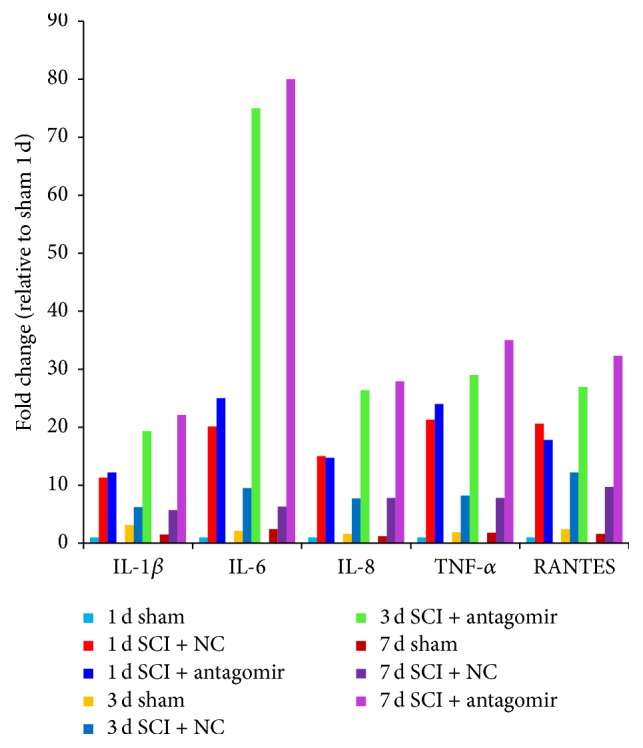
Inflammatory cytokines levels of different model groups. Every inflammatory cytokine was detected in 3 time points (1 day, 3 days, and 7 days after SCI). The same color represents the same treatment at the same time between different cytokines. The expression levels are shown as the mean ± SD.

Compared to the sham animals, the proinflammatory cytokines, such as IL-1, IL-6, IL-8, TNF-*α*, and RANTES, were increased on the first day after SCI surgery but reduced 3 or 7 days after SCI (SCI + NC). However, the proinflammatory cytokines kept increasing when the animals were injected with miRNA-146a antagomir (SCI + antagomir) (*P* < 0.05, Figure 6). In this sense, inhibition of miRNA-146a could positively regulate proinflammatory cytokines in the rat SCI model.

### 3.4. Recovery of SCI and BBB

**Figure 7:**
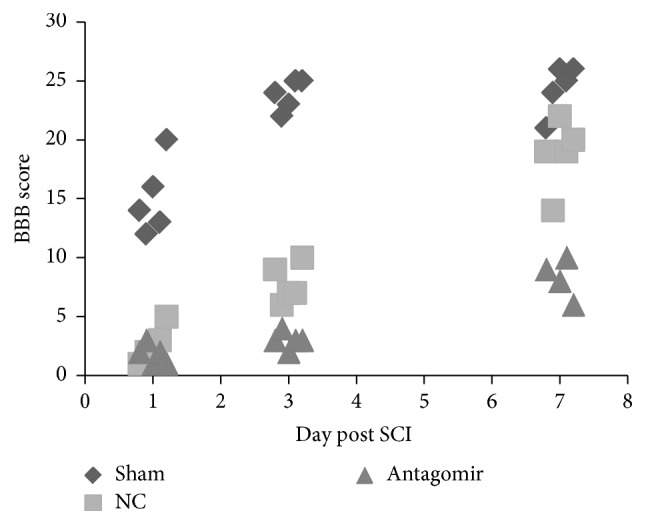
BBB scores of different model groups. 5 BBB scores were adopted 1 day and 3 days after SCI; 4 BBB scores were adopted, while only 4 animals were left 7 days after SCI. The expression levels are shown as the mean ± SD.

The BBB scores showed that athletic ability was recovered in all the sham animals 3 days after SCI surgery. However, animals in the SCI + NC group recovered slowly compared to the sham group, and they regained athletic ability 7 days after SCI. Animals in the SCI + antagomir group endured more during the experiment, and even on day 7, the BBB scores were only approximately 5~10. Additionally, one of the animals in the antagomir group died on the 5th day after SCI surgery (Figure 7).

## 4. Discussion

Accumulating evidence suggests that miRNA-146a acts as a modulator of the innate immune response and affects the inflammatory cytokine levels in immunological and other brain cell types. Lukiw recently reported that an upregulated miRNA-146a might be integral to innate immune or inflammatory brain cell responses in prion-mediated infections and to progressive and irreversible neurodegeneration of both the murine and human brain [23]. Although accumulated evidence suggests that miRNA-146a may participate in SCI recovery [5, 8, 24], there is still limited knowledge of the miRNA-146a regulatory networks that modulate genes expression.

*IRAK1* and* TRAF6* are known to be part of the common signaling pathway of the TIR super family, and they are also involved in the MAPK pathway. However, although several studies have reported that* IRAK1* and* TRAF6 *were downregulated following miRNA-146a overexpression [14–18], the overexpression did not appear to be responsible for the responses that were observed following SCI recovery.

In our study, we found that* IRAK1* and* TRAF6 *transcription in the SCI + NC group was upregulated on the first day after surgery compared to the sham group and then that transcription was significantly downregulated 3 and 7 days after the surgery (Figure 3). Similarly, the secretion of proinflammatory cytokines, including IL-6, IL-8, TNF-*α*, and RANTES, was extremely upregulated on the first day after surgery, whereas their expression was significantly downregulated 3 and 7 days after surgery in the SCI + NC group. Furthermore, the secretions of proinflammatory cytokines, as well as the protein levels of the two genes, rose sharply after antagomir was injected into the animals (Figures 4 and 6). Conversely, miRNA-146a expression was upregulated 3 and 7 days after surgery, whereas no remarkable expression differences were observed on the first day after surgery (Figure 2) in the SCI + NC group. Our data suggested that miRNA-146a played a repressive role in* IRAK1* and* TRAF6* expression and subsequently downregulated proinflammation cytokines. This outcome was consistent with a former study that reported that miRNA-146a inhibited the activation of NF-*κ*B [25] and the expression of some other inflammatory mediators, including TNF-a, IL-6, COX-2, and CXCL12 [15, 26, 27], which suggests an anti-inflammatory effect for miRNA-146a.

Moreover, the former study indicated that miRNA-146a negatively regulated the inflammatory response in human gingival fibroblasts by directly binding to the* IRAK1* 3′-UTR [28]. Also, the bioinformatic analysis exhibited that there are binding sites in the* TRAF6* 3′ UTR. Our luciferase assay also indicated that miRNA-146a could repress luciferase expression to directly target the 3′ UTR of* TRAF6* and* IRAK1* (Figure 5). Furthermore, the expression of* TRAF6* and* IRAK1 *rose sharply after antagomir was injected into the animal model. Therefore, we speculated that miRNA-146a suppressed the expression of* TRAF6* and* IRAK1* via binding to the 3′ UTR. Consistent with this outcome, our data demonstrated that miRNA-146a could suppress the inflammation induced by SCI through downregulating* IRAK1* and* TRAF6*.

**Figure 8:**
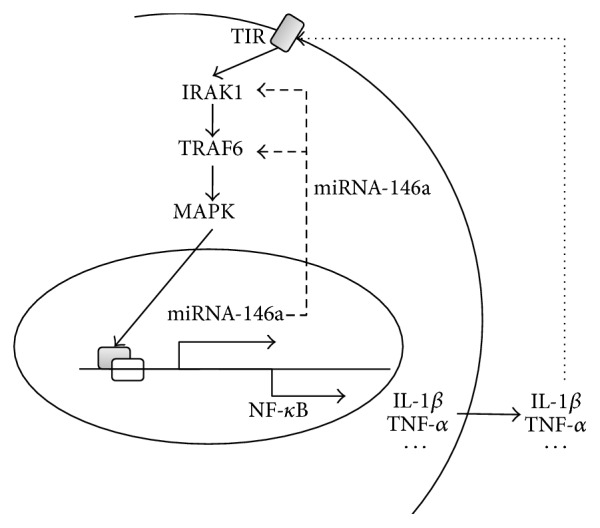
Schematic of miR-146a regulating inflammation by a negative feedback mechanism in the SCI.

The inflammatory response is usually modulated by molecular immune mediators, such as inflammatory cytokines (TNF-a, IL-6, etc.). Usually, excess proinflammatory cytokines would enhance the inflammation signaling pathway and induce more proinflammatory cytokines. This increase would initiate a vicious feedback loop that may play important roles in inducing or maintaining long-term inflammation (Figure 8).

However, our present data showed that SCI did not change miRNA-146a expression in the early phase (1 day), but the expression of miRNA-146a significantly increased in the maintenance phase (3 and 7 days). This result implied that the upregulation of miRNA-146a might be activated by proinflammation cytokines. In fact, Nakasa et al. [29] reported that TNF*α* and IL-1*β* could induce miRNA-146a expression in human rheumatoid arthritis synovial fibroblasts. Taganov et al. have also shown that miRNA-146a can be induced by NF-*κ*B activation [16].

Furthermore, Xie et al. reported that miRNA-146a, IL-1, IL-6, and TNF-*α* levels were downregulated when inhibiting NF-*κ*B. But, on the contrary, p50/p65 strongly activated the miRNA-146a promoter in a luciferase assay [30]. Wang et al. [17] found that intrathecal injection of amiRNA-146a mimic might exert antinociceptive effects by regulating the* TRAF6* and* IRAK1 *function in the DRG.

Therefore, we speculated that miRNA-146a could not only contribute to SCI recovery through inhibition of the* IRAK1/TRAF6* pathway but also could be induced by proinflammatory cytokines downstream of the* IRAK1/TRAF6 *signaling pathway (Figure 8). This feedback mechanism provided an efficient way to avoid the severe inflammatory response induced by SCI, which contributed to the recovery of SCI.

These findings collectively indicate that miRNA-146a upregulation in SCI may be driven by proinflammatory cytokines, which in turn negatively regulate the proinflammatory cytokines by downregulating the expression of* TRAF6* and* IRAK1* [31]. Thus, overexpression of miRNA-146a at 7 days after surgery for SCI may be a consequence of an increase in proinflammatory cytokines on previous time (1 day). Then the upregulation of miRNA-146a may have in turn inactivated this pathway via a negative feedback mechanism so that the* IRAK1/TRAF6* pathway would not be overactivated. Therefore, miRNA-146a may serve as a powerful potential therapeutic target for SCI. However, we did not test whether an overdose of miR-146a mimic could improve the motor function after SCI, as the miRNA-146a level was already greatly upregulated (about 4 times), and the extra overdose of miR-146a mimic would overaffect the inflammatory reaction, which may be possibly harmful to the SCI recovery. But we do not disavow the possibility that overdose of miR-146a mimic may improve the motor function after SCI, which should be certified by further experiment.

## Figures and Tables

**Table 1:** Primers used in the study.

Primer	Sequence
TRAF6 forward	TCATTATGATCTGGACTGCCCAAC
TRAF6 reverse	TGCAAGTGTCGTGCCAAGTG
IRAK1 forward	GCCCGAGGAGTACATCAAGA
IRAK1 reverse	CTCTGACCAGCCAAGGTCTC
GAPDH forward	TGTTCCTACCCCCAATGTG
GAPDH reverse	GTGTAGCCCAAGATGCCCT
miR-146a-5p forward	TGAGAACTGAATTCCATGGGT
U6 forward	GCTTCGGCAGCACATATACTAA
U6 reverse	CGAATTTGCGTGTCATCCTT

## References

[1] Bareyre F. M., Schwab M. E. (2003). Inflammation, degeneration and regeneration in the injured spinal cord: insights from DNA microarrays. *Trends in Neurosciences*.

[2] Profyris C., Cheema S. S., Zang D., Azari M. F., Boyle K., Petratos S. (2004). Degenerative and regenerative mechanisms governing spinal cord injury. *Neurobiology of Disease*.

[3] Nesic O., Svrakic N. M., Xu G.-Y. (2002). DNA microarray analysis of the contused spinal cord: effect of NMDA receptor inhibition. *Journal of Neuroscience Research*.

[4] De Biase A., Knoblach S. M., Di Giovanni S. (2005). Gene expression profiling of experimental traumatic spinal cord injury as a function of distance from impact site and injury severity. *Physiological Genomics*.

[5] Yunta M., Nieto-Díaz M., Esteban F. J. (2012). MicroRNA dysregulation in the spinal cord following traumatic injury. *PloS ONE*.

[6] Alvarez-Garcia I., Miska E. A. (2005). MicroRNA functions in animal development and human disease. *Development*.

[7] Zamore P. D., Haley B. (2005). Ribo-gnome: the big world of small RNAs. *Science*.

[8] Strickland E. R., Hook M. A., Balaraman S., Huie J. R., Grau J. W., Miranda R. C. (2011). MicroRNA dysregulation following spinal cord contusion: implications for neural plasticity and repair. *Neuroscience*.

[9] Lindsay M. A. (2008). microRNAs and the immune response. *Trends in Immunology*.

[10] Sonkoly E., Pivarcsi A. (2009). microRNAs in inflammation. *International Reviews of Immunology*.

[11] Taganov K. D., Boldin M. P., Chang K.-J., Baltimore D. (2006). NF-*κ*B-dependent induction of microRNA miR-146, an inhibitor targeted to signaling proteins of innate immune responses. *Proceedings of the National Academy of Sciences of the United States of America*.

[12] Cui J. G., Li Y. Y., Zhao Y., Bhattacharjee S., Lukiw W. J. (2010). Differential regulation of interleukin-1 receptor-associated kinase-1 (IRAK-1) and IRAK-2 by microRNA-146a and NF-*κ*B in stressed human astroglial cells and in Alzheimer disease. *The Journal of Biological Chemistry*.

[13] Lukiw W. J., Zhao Y., Jian G. C. (2008). An NF-*κ*B-sensitive micro RNA-146a-mediated inflammatory circuit in alzheimer disease and in stressed human brain cells. *The Journal of Biological Chemistry*.

[14] Hou J., Wang P., Lin L. (2009). MicroRNA-146a feedback inhibits RIG-I-dependent Type I IFN production in macrophages by targeting *TRAF6, IRAK1*, and *IRAK2*. *The Journal of Immunology*.

[15] Iyer A., Zurolo E., Prabowo A. (2012). MicroRNA-146a: a key regulator of astrocyte-mediated inflammatory response. *PLoS ONE*.

[16] Taganov K. D., Boldin M. P., Chang K.-J., Baltimore D. (2006). NF-*κ*B-dependent induction of microRNA miR-146, an inhibitor targeted to signaling proteins of innate immune responses. *Proceedings of the National Academy of Sciences of the United States of America*.

[17] Wang L., Chopp M., Szalad A. (2014). The role of miR-146a in dorsal root ganglia neurons of experimental diabetic peripheral neuropathy. *Neuroscience*.

[18] Lu Y., Cao D.-L., Jiang B.-C., Yang T., Gao Y.-J. (2015). MicroRNA-146a-5p attenuates neuropathic pain via suppressing *TRAF6* signaling in the spinal cord. *Brain, Behavior, and Immunity*.

[19] Liu N.-K., Wang X.-F., Lu Q.-B., Xu X.-M. (2009). Altered microRNA expression following traumatic spinal cord injury. *Experimental Neurology*.

[20] Lu Y., Jiang B.-C., Cao D.-L. (2014). TRAF6 upregulation in spinal astrocytes maintains neuropathic pain by integrating TNF-*α* and IL-1*β* signaling. *Pain*.

[21] Li Y. Y., Cui J. G., Dua P., Pogue A. I., Bhattacharjee S., Lukiw W. J. (2011). Differential expression of miRNA-146a-regulated inflammatory genes in human primary neural, astroglial and microglial cells. *Neuroscience Letters*.

[22] Basso D. M., Beattie M. S., Bresnahan J. C. (1995). A sensitive and reliable locomotor rating scale for open field testing in rats. *Journal of Neurotrauma*.

[23] Lukiw W. J., Dua P., Pogue A. I., Eicken C., Hill J. M. (2011). Upregulation of micro RNA-146a (miRNA-146a), a marker for inflammatory neurodegeneration, in sporadic creutzfeldt-jakob disease (sCJD) and gerstmann-straussler-scheinker (GSS) syndrome. *Journal of Toxicology and Environmental Health—Part A*.

[24] Lukiw W. J., Dua P., Pogue A. I., Eicken C., Hill J. M. (2011). Upregulation of micro RNA-146a (miRNA-146a), a marker for inflammatory neurodegeneration, in sporadic creutzfeldt-jakob disease (sCJD) and gerstmann-straussler-scheinker (GSS) syndrome. *Journal of Toxicology and Environmental Health–Part A: Current Issues*.

[25] Paik J. H., Jang J.-Y., Jeon Y. K. (2011). MicroRNA-146a downregulates NF*κ*B activity via targeting TRAF6 and functions as a tumor suppressor having strong prognostic implications in NK/T cell lymphoma. *Clinical Cancer Research*.

[26] Li S., Yue Y., Xu W., Xiong S. (2013). MicroRNA-146a represses mycobacteria-induced inflammatory response and facilitates bacterial replication via targeting IRAK-1 and TRAF-6. *PLoS ONE*.

[27] Li X., Gibson G., Kim J.-S. (2011). MicroRNA-146a is linked to pain-related pathophysiology of osteoarthritis. *Gene*.

[28] Xie Y.-F., Shu R., Jiang S.-Y., Liu D.-L., Ni J., Zhang X.-L. (2013). MicroRNA-146 inhibits pro-inflammatory cytokine secretion through IL-1 receptor-associated kinase 1 in human gingival fibroblasts. *Journal of Inflammation*.

[29] Nakasa T., Miyaki S., Okubo A. (2008). Expression of microRNA-146 in rheumatoid arthritis synovial tissue. *Arthritis & Rheumatism*.

[30] Xie Y., Shu R., Jiang S. (2014). miRNA-146 negatively regulates the production of pro-inflammatory cytokines via NF-*κ*B signalling in human gingival fibroblasts. *Journal of Inflammation*.

[31] Ma X., Becker Buscaglia L. E., Barker J. R., Li Y. (2011). MicroRNAs in NF-*κ*B signaling. *Journal of Molecular Cell Biology*.

